# Supplementary material for: Design deep neural network architecture using a genetic algorithm for estimation of pile bearing capacity
Source: PLoS One. 2020 Dec 17;15(12):e0243030. doi: 10.1371/journal.pone.0243030 (PMC7746167; doi:10.1371/journal.pone.0243030)
Supplement: S1 Appendix — (DOCX) [file pone.0243030.s002.docx]

**S1 Appendix**

**S1 Table A.** An example of data set of the present study.

| **N°** | **D** | **Z_1_** | **Z_2_** | **Z_3_** | **Z_p_** | **Z_g_** | **Z_t_** | **Z_m_** | **N_sh_** | **N_t_** | **P_u_** |
| --- | --- | --- | --- | --- | --- | --- | --- | --- | --- | --- | --- |
| Unit | mm | M | m | m | m | m | m | m | - | - | kN |
| 1 | 400 | 3.45 | 8 | 0.14 | 2.95 | 3.52 | 2.98 | 14.54 | 11.59 | 7.48 | 885 |
| 2 | 400 | 3.4 | 7.3 | 0 | 3.4 | 3.61 | 3.51 | 14.1 | 10.7 | 7.28 | 1115.2 |
| 3 | 400 | 4.25 | 8 | 0.2 | 2.15 | 3.55 | 2.95 | 14.6 | 12.45 | 7.20 | 1392 |
| 4 | 400 | 3.4 | 7.31 | 0 | 3.4 | 3.54 | 3.43 | 14.11 | 10.71 | 7.28 | 1032.8 |
| 5 | 400 | 3.45 | 6.29 | 0 | 3.35 | 3.44 | 3.35 | 13.09 | 9.74 | 7.02 | 1240 |
| 6 | 400 | 4.35 | 8 | 0.8 | 2.05 | 3.45 | 2.25 | 15.2 | 13.15 | 7.52 | 1392 |
| 7 | 400 | 3.45 | 8 | 0.22 | 2.95 | 3.57 | 2.95 | 14.62 | 11.67 | 7.53 | 1318 |
| 8 | 400 | 4.1 | 2.01 | 0 | 2.7 | 3.53 | 2.72 | 8.81 | 6.11 | 4.80 | 528 |
| 9 | 400 | 3.4 | 7.24 | 0 | 3.4 | 3.44 | 3.4 | 14.04 | 10.64 | 7.26 | 967 |
| 10 | 400 | 4.35 | 8 | 1.11 | 2.05 | 3.56 | 2.05 | 15.51 | 13.46 | 7.69 | 1128.6 |
| 11 | 400 | 3.4 | 7.4 | 0 | 3.4 | 3.61 | 3.41 | 14.2 | 10.8 | 7.30 | 1088.8 |
| 12 | 400 | 4.35 | 8 | 0.3 | 2.05 | 3.45 | 2.75 | 14.7 | 12.65 | 7.22 | 1473 |
| 13 | 300 | 3.4 | 5.25 | 0 | 3.4 | 3.47 | 3.42 | 12.05 | 8.65 | 6.75 | 585.4 |
| 14 | 400 | 4.75 | 7.25 | 0 | 2.05 | 3.65 | 3.6 | 14.05 | 12 | 6.73 | 1425 |
| 15 | 400 | 4.25 | 8 | 0.4 | 2.15 | 3.59 | 2.79 | 14.8 | 12.65 | 7.32 | 1551 |
| 16 | 300 | 3.4 | 5.25 | 0 | 3.4 | 3.48 | 3.43 | 12.05 | 8.65 | 6.75 | 508.9 |
| 17 | 400 | 3.45 | 8 | 0.3 | 2.95 | 3.65 | 2.95 | 14.7 | 11.75 | 7.59 | 1017.9 |
| 18 | 300 | 3.4 | 5.2 | 0 | 3.4 | 3.41 | 3.41 | 12 | 8.6 | 6.73 | 610.7 |
| 19 | 400 | 4.25 | 8 | 0.4 | 2.15 | 3.55 | 2.75 | 14.8 | 12.65 | 7.32 | 1392 |
| 20 | 400 | 4.1 | 2.08 | 0 | 2.7 | 3.58 | 2.7 | 8.88 | 6.18 | 4.86 | 480 |
| 21 | 400 | 4.65 | 7.5 | 0 | 2.15 | 3.55 | 3.25 | 14.3 | 12.15 | 6.82 | 1551 |
| 22 | 400 | 4.1 | 2.08 | 0 | 2.7 | 3.63 | 2.75 | 8.88 | 6.18 | 4.86 | 432 |
| 23 | 400 | 3.55 | 5.36 | 0 | 3.25 | 3.41 | 3.25 | 12.16 | 8.91 | 6.71 | 930 |
| 24 | 400 | 4.35 | 8 | 0.95 | 2.05 | 3.41 | 2.06 | 15.35 | 13.3 | 7.60 | 1323.2 |
| 25 | 400 | 4.35 | 8 | 1.05 | 2.05 | 3.5 | 4.35 | 15.45 | 13.4 | 7.66 | 1297.8 |
| 26 | 400 | 4.35 | 8 | 1.07 | 2.05 | 3.52 | 2.05 | 15.47 | 13.42 | 7.67 | 1082.3 |
| 27 | 300 | 3.4 | 5.2 | 0 | 3.4 | 3.43 | 3.43 | 12 | 8.6 | 6.73 | 600.7 |
| 28 | 300 | 3.4 | 5.25 | 0 | 3.4 | 3.46 | 3.41 | 12.05 | 8.65 | 6.75 | 407.2 |
| 29 | 300 | 3.4 | 5.2 | 0 | 3.4 | 3.42 | 3.42 | 12 | 8.6 | 6.73 | 660.6 |
| 30 | 400 | 3.45 | 8 | 0.3 | 2.95 | 3.66 | 2.96 | 14.7 | 11.75 | 7.59 | 960 |
| 31 | 400 | 3.5 | 8 | 0.17 | 2.9 | 3.47 | 2.9 | 14.57 | 11.67 | 7.48 | 960 |
| 32 | 400 | 3.4 | 7.33 | 0 | 3.4 | 3.55 | 3.42 | 14.13 | 10.73 | 7.28 | 1094.25 |
| 33 | 400 | 3.45 | 8 | 0.25 | 2.95 | 3.6 | 2.95 | 14.65 | 11.7 | 7.55 | 960 |
| 34 | 400 | 4.65 | 7.5 | 0 | 2.15 | 3.59 | 3.29 | 14.3 | 12.15 | 6.82 | 1551 |
| 35 | 400 | 3.4 | 7.35 | 0 | 3.4 | 3.56 | 3.41 | 14.15 | 10.75 | 7.29 | 1052.4 |
| 36 | 400 | 3.4 | 7.28 | 0 | 3.4 | 3.48 | 3.4 | 14.08 | 10.68 | 7.27 | 1318 |
| 37 | 400 | 3.55 | 5.39 | 0 | 3.25 | 3.44 | 3.25 | 12.19 | 8.94 | 6.72 | 1083 |
| 38 | 400 | 4.1 | 2 | 0 | 2.7 | 3.56 | 2.76 | 8.8 | 6.1 | 4.80 | 620 |
| 39 | 400 | 4.35 | 8 | 0.7 | 2.05 | 3.49 | 2.39 | 15.1 | 13.05 | 7.46 | 1392 |
| 40 | 300 | 3.4 | 5.2 | 0 | 3.4 | 3.43 | 3.43 | 12 | 8.6 | 6.73 | 559.8 |
| 41 | 400 | 4.35 | 8 | 1.01 | 2.05 | 3.46 | 2.05 | 15.41 | 13.36 | 7.64 | 1550 |
| 42 | 400 | 4.35 | 8 | 1.06 | 2.05 | 3.55 | 2.09 | 15.46 | 13.41 | 7.66 | 1321 |
| 43 | 400 | 4.35 | 8 | 0.05 | 2.05 | 3.58 | 3.13 | 14.45 | 12.4 | 7.07 | 1344 |
| 44 | 400 | 4.05 | 8 | 0.66 | 2.35 | 3.46 | 2.4 | 15.06 | 12.71 | 7.56 | 1318 |
| 45 | 300 | 3.4 | 5.23 | 0 | 3.4 | 3.44 | 3.41 | 12.03 | 8.63 | 6.74 | 585.35 |
| 46 | 400 | 5.4 | 6.3 | 0 | 2.15 | 3.52 | 1.06 | 13.1 | 14.7 | 5.50 | 1056 |
| 47 | 400 | 3.45 | 8 | 0.19 | 2.95 | 3.56 | 2.97 | 14.59 | 11.64 | 7.52 | 1318 |
| 48 | 400 | 4.45 | 7.21 | 0 | 2.35 | 3.41 | 2.4 | 14.01 | 11.66 | 6.83 | 1318 |
| 49 | 400 | 4.35 | 8 | 1.05 | 2.05 | 3.53 | 2.08 | 15.45 | 13.4 | 7.66 | 1473 |
| 50 | 400 | 4.1 | 1.9 | 0 | 2.7 | 3.43 | 2.73 | 8.7 | 6 | 4.72 | 620 |
| 51 | 400 | 3.85 | 7.5 | 0 | 2.95 | 3.68 | 3.38 | 14.3 | 11.35 | 7.13 | 1425 |
| 52 | 400 | 4.35 | 8 | 0.95 | 2.05 | 3.44 | 2.09 | 15.35 | 13.3 | 7.60 | 1152 |
| 53 | 400 | 4.35 | 8 | 1.18 | 2.05 | 3.66 | 2.08 | 15.58 | 13.53 | 7.73 | 1056 |
| 54 | 400 | 4.35 | 8 | 1.04 | 2.05 | 3.52 | 2.08 | 15.44 | 13.39 | 7.65 | 1321 |
| 55 | 400 | 4.1 | 2 | 0 | 2.7 | 3.54 | 2.74 | 8.8 | 6.1 | 4.80 | 712.5 |
| 56 | 400 | 3.4 | 7.3 | 0 | 3.4 | 3.5 | 3.4 | 14.1 | 10.7 | 7.28 | 900 |
| 57 | 400 | 4.35 | 8 | 1 | 2.05 | 3.45 | 2.05 | 15.4 | 13.35 | 7.63 | 1119.7 |
| 58 | 400 | 3.45 | 8 | 0.12 | 2.95 | 3.47 | 2.95 | 14.52 | 11.57 | 7.47 | 1318 |
| 59 | 400 | 4.65 | 7.35 | 0 | 2.15 | 3.55 | 3.4 | 14.15 | 12 | 6.79 | 1392 |
| 60 | 400 | 4.35 | 8 | 0.98 | 2.05 | 3.48 | 2.1 | 15.38 | 13.33 | 7.62 | 1224.8 |
| 61 | 400 | 3.85 | 7.3 | 0 | 2.95 | 3.68 | 3.58 | 14.1 | 11.15 | 7.08 | 1440 |
| 62 | 300 | 3.4 | 5.25 | 0 | 3.4 | 3.46 | 3.41 | 12.05 | 8.65 | 6.75 | 661.6 |
| 63 | 400 | 3.4 | 7.23 | 0 | 3.4 | 3.43 | 3.4 | 14.03 | 10.63 | 7.26 | 960 |
| 64 | 400 | 4.1 | 1.8 | 0 | 2.7 | 3.39 | 2.79 | 8.6 | 5.9 | 4.64 | 620 |
| 65 | 400 | 4.35 | 8 | 1.05 | 2.05 | 3.55 | 2.1 | 15.45 | 13.4 | 7.66 | 1221.5 |
| 66 | 400 | 4.35 | 8 | 1.01 | 2.05 | 3.46 | 2.05 | 15.41 | 13.36 | 7.64 | 1473 |
| 67 | 400 | 3.4 | 7.3 | 0 | 3.4 | 3.54 | 3.44 | 14.1 | 10.7 | 7.28 | 967 |
| 68 | 400 | 4.35 | 8 | 1.07 | 2.05 | 3.52 | 2.05 | 15.47 | 13.42 | 7.67 | 1425 |
| 69 | 400 | 4.35 | 8 | 1.08 | 2.05 | 3.53 | 2.05 | 15.48 | 13.43 | 7.67 | 1248 |
| 70 | 400 | 4.75 | 7.4 | 0 | 2.05 | 3.52 | 3.32 | 14.2 | 12.15 | 6.76 | 1425 |
| 71 | 300 | 3.4 | 5.22 | 0 | 3.4 | 3.44 | 3.42 | 12.02 | 8.62 | 6.74 | 617 |
| 72 | 400 | 4.35 | 8 | 0.96 | 2.05 | 3.42 | 2.06 | 15.36 | 13.31 | 7.61 | 1244 |
| 73 | 400 | 3.45 | 5.24 | 0 | 3.35 | 3.44 | 3.4 | 12.04 | 8.69 | 6.72 | 1240 |
| 74 | 400 | 4.35 | 8 | 1.03 | 2.05 | 3.48 | 2.05 | 15.43 | 13.38 | 7.65 | 1318 |
| 75 | 400 | 4.45 | 8 | 1.18 | 1.95 | 3.58 | 2 | 15.58 | 13.63 | 7.69 | 1032.4 |
| 76 | 400 | 4.75 | 7.6 | 0 | 2.05 | 3.49 | 3.09 | 14.4 | 12.35 | 6.81 | 1473 |
| 77 | 400 | 3.4 | 7.3 | 0 | 3.4 | 3.49 | 3.39 | 14.1 | 10.7 | 7.28 | 1068.8 |
| 78 | 400 | 3.4 | 7.31 | 0 | 3.4 | 3.56 | 3.45 | 14.11 | 10.71 | 7.28 | 1032.4 |
| 79 | 400 | 4.25 | 8 | 0.1 | 2.15 | 3.54 | 3.04 | 14.5 | 12.35 | 7.14 | 1551 |
| 80 | 400 | 4.75 | 7.5 | 0 | 2.05 | 3.6 | 3.3 | 14.3 | 12.25 | 6.79 | 1425 |
| 81 | 400 | 4.75 | 7.5 | 0 | 2.05 | 3.45 | 3.15 | 14.3 | 12.25 | 6.79 | 1297.8 |
| 82 | 400 | 4.1 | 1.72 | 0 | 2.7 | 3.27 | 2.75 | 8.52 | 5.82 | 4.57 | 423.9 |
| 83 | 400 | 3.45 | 8 | 0.1 | 2.95 | 3.54 | 3.04 | 14.5 | 11.55 | 7.46 | 1017.9 |
| 84 | 400 | 4.35 | 8 | 1.1 | 2.05 | 3.55 | 2.05 | 15.5 | 13.45 | 7.69 | 1425 |
| 85 | 400 | 4.35 | 8 | 0.95 | 2.05 | 3.41 | 2.06 | 15.35 | 13.3 | 7.60 | 1110.6 |
| 86 | 400 | 4.05 | 8 | 0.7 | 2.35 | 3.47 | 2.37 | 15.1 | 12.75 | 7.58 | 1318 |
| 87 | 400 | 3.45 | 8 | 0.07 | 2.95 | 3.42 | 2.95 | 14.47 | 11.52 | 7.44 | 1240 |
| 88 | 400 | 4.05 | 8 | 0.7 | 2.35 | 3.48 | 2.38 | 15.1 | 12.75 | 7.58 | 1238 |
| 89 | 400 | 4.75 | 7.25 | 0 | 2.05 | 3.62 | 3.57 | 14.05 | 12 | 6.73 | 1425 |
| 90 | 400 | 3.45 | 8 | 0.2 | 2.95 | 3.52 | 2.92 | 14.6 | 11.65 | 7.52 | 967 |
| 91 | 400 | 3.5 | 8 | 0.2 | 2.9 | 3.51 | 2.91 | 14.6 | 11.7 | 7.50 | 960 |
| 92 | 400 | 3.4 | 7.3 | 0 | 3.4 | 3.5 | 3.4 | 14.1 | 10.7 | 7.28 | 958 |
| 93 | 300 | 3.4 | 5.2 | 0 | 3.4 | 3.48 | 3.48 | 12 | 8.6 | 6.73 | 611.6 |
| 94 | 400 | 4.35 | 8 | 0.97 | 2.05 | 3.42 | 2.05 | 15.37 | 13.32 | 7.61 | 1317 |
| 95 | 400 | 3.85 | 7.35 | 0 | 2.95 | 3.64 | 3.49 | 14.15 | 11.2 | 7.09 | 1425 |
| 96 | 400 | 4.75 | 7.6 | 0 | 2.05 | 3.44 | 3.04 | 14.4 | 12.35 | 6.81 | 1473 |
| 97 | 400 | 4.65 | 7.4 | 0 | 2.15 | 3.59 | 3.39 | 14.2 | 12.05 | 6.80 | 1551 |
| 98 | 400 | 4.35 | 8 | 1.02 | 2.05 | 3.47 | 4.05 | 15.42 | 13.37 | 7.64 | 1318 |
| 99 | 400 | 3.45 | 8 | 0.14 | 2.95 | 3.52 | 2.98 | 14.54 | 11.59 | 7.48 | 885 |
| 100 | 400 | 3.4 | 7.3 | 0 | 3.4 | 3.61 | 3.51 | 14.1 | 10.7 | 7.28 | 1115.2 |
